# The Way to Ultrafast, High-Throughput Enantioseparations of Bioactive Compounds in Liquid and Supercritical Fluid Chromatography

**DOI:** 10.3390/molecules23102709

**Published:** 2018-10-20

**Authors:** Omar H. Ismail, Simona Felletti, Chiara De Luca, Luisa Pasti, Nicola Marchetti, Valentina Costa, Francesco Gasparrini, Alberto Cavazzini, Martina Catani

**Affiliations:** 1 Department of Drug Chemistry and Technology, “Sapienza” University of Rome, P. le Aldo Moro 5, 00185 Rome, Italy; omar.ismail@uniroma1.it (O.H.I.); francesco.gasparrini@uniroma1.it (F.G.); 2 Department of Chemistry and Pharmaceutical Sciences, University of Ferrara, via L. Borsari 46, 44121 Ferrara, Italy; simona.felletti@unife.it (S.F.); chiara.deluca@unife.it (C.D.L.); psu@unife.it (L.P.); nicola.marchetti@unife.it (N.M.); valentina.costa@unife.it (V.C.); cvz@unife.it (A.C.)

**Keywords:** ultra-high performance liquid chromatography (UHPLC), supercritical fluid chromatography (SFC), ultrafast enantioseparations, bioactive chiral compounds

## Abstract

Until less than 10 years ago, chiral separations were carried out with columns packed with 5 or 3 μm fully porous particles (FPPs). Times to resolve enantiomeric mixtures were easily larger than 30 min, or so. Pushed especially by stringent requirements from medicinal and pharmaceutical industries, during the last years the field of chiral separations by liquid chromatography has undergone what can be defined a “true revolution”. With the purpose of developing ever faster and efficient method of separations, indeed, very efficient particle formats, such as superficially porous particles (SPPs) or sub-2 μm FPPs, have been functionalized with chiral selectors and employed in ultrafast applications. Thanks to the use of short column (1–2 cm long), packed with these extremely efficient chiral stationary phases (CSPs), operated at very high flow rates (5–8 mL/min), resolution of racemates could be accomplished in very short time, in many cases less than 1 s in normal-, reversed-phase and HILIC conditions. These CSPs have been found to be particularly promising also to carry out high-throughput separations under supercritical fluid chromatography (SFC) conditions. The most important results that have been recently achieved in terms of ultrafast, high-throughput enantioseparations both in liquid and supercritical fluid chromatography with particular attention to the very important field of bioactive chiral compounds will be reviewed in this manuscript. Attention will be focused not only on the latest introduced CSPs and their applications, but also on instrumental modifications which are required in some cases in order to fully exploit the intrinsic potential of new generation chiral columns.

## 1. Introduction

Separation of chiral compounds has always been one of the most challenging applications of liquid chromatography (LC). Since enantiomers have the same chemo-physical properties, conventional approaches used to separate achiral molecules cannot be adopted for chiral separations. For this reason, for over thirty years, research activities in the field of LC enantioseparations have been essentially focused on the development of novel chiral stationary phases (CSPs), able to resolve the largest number of enantiomeric pairs. Different CSPs have been, thus, developed by functionalizing 3 or 5 μm fully porous particles (FPPs) with different chiral selectors including polysaccharides, macrocyclic glycopeptides, Pirkle-type ones, ion-exchangers, cyclofructans [1]. However, during the last ten years the attention of scientists has been moved from the research for ever better enantioselectivity to the efficiency of enantioseparations and, most importantly, speed factors [2,3]. This change of direction has been essentially driven by pressing requirements from medicinal and pharmaceutical industries for the development of faster and more efficient methods. Indeed, elution of enantiomers from chiral columns packed with 3 or 5 μm FPPs typically requires 5 to 30 min at least. Since chiral separations are fundamental in many stages of drug development (i.e., high-throughput screenings of large libraries of chiral molecules, monitoring steps), it follows that they could represent a time-consuming and limiting step of the production process, if too slow.

In the last few years, technological progress in materials and particle manufacturing has allowed to introduce, in chiral chromatography, very efficient particle formats, such as sub-2 μm FPPs and second-generation superficially porous particles (SPPs), routinely used as stationary phases for achiral reversed-phase (RP) C${}_{18}$ columns. At early stages, improvements in preparation of CSPs have been limited for many years essentially by the following reasons: (i) the complex adaptation of surface modification methods from larger to smaller particles; (ii) the tendency of small particles to aggregation during chemical modification; (iii) low mechanical resistance and stability of small particles functionalized with chiral selectors when operated at high flow rates/pressures; (iv) the lack of fundamental studies in chiral LC [4].

The introduction of packed columns made of new generation particles has represented the most important innovation in the field of chiral LC, allowing to reach efficiencies comparable to those typical of RP achiral separations (in the order of 300,000 plates/m) [5,6,7]. Thanks to the very good kinetic performance of these particles, it is possible to increase the flow rate without remarkable loss of efficiency, opening the way to fast (or ultrafast) high-throughput enantioseparations.

The first report on the use of sub-2 μm FPPs in chiral LC was published in 2010 by Gasparrini and coworkers [8,9,10,11]. They packed columns with 1.9 μm FPPs functionalized with a Pirkle-type chiral selector that allowed to obtain the separation of different pairs of enantiomers in a range of 15–40 s. In 2011, Lindner and coworkers prepared the first CSP by using second-generation SPPs functionalized with a weak anion exchanger chiral selector [12]. During the following year, Chankvetadze et al. published the first work in which sub-2 μm FPPs and sub-3 μm SPPs, functionalized with the same polysaccharide chiral selector, were compared [13]. The most comprehensive work aimed at investigating kinetic performance of columns packed with both sub-2 μm FPPs and second-generation SPPs is that by Armstrong’s group. They compared CSPs based on different chiral selectors (i.e., polysaccharides, cyclofructans, macrocyclic glycopeptides) operated in different chromatographic modes, including RP, hydrophilic interaction chromatography (HILIC), polar organic mode (POM) [2,14,15,16,17,18]. Results of these investigations have shown that chiral SPPs outperform their FP counterparts in every chromatographic mode. Following these authors, the reasons behind the better performance of chiral SPPs are the same as those already extensively demonstrated for achiral hydrophobic SPPs [19], namely a reduced longitudinal diffusion, smaller solid-liquid mass transfer resistance and better packing quality (hence, smaller eddy dispersion).

Even though it is true that SPPs, due to their geometrical characteristics, allow for the reduction of all those effects associated with diffusion inside particles, the same concepts cannot be taken for granted in chiral chromatography. By comparing columns packed with FPPs (2.5 and 1.8 μm) and SPPs (2.6 μm) functionalized with Whelk-O1 chiral selector operated in normal phase mode, for instance, some of the authors of this work found that, unexpectedly, the column packed with the latter CSP provide worse kinetic performance than FPP ones [6]. This has been related essentially to two main reasons [4]. The first one is an higher contribution of eddy dispersion, most likely coming from some difficulties encountered during the packing process of chiral SPPs. This suggests that slurry packing polar SPPs (such as chiral ones) could be more difficult than for hydrophobic ones for which, on the other hand, it has been largely demonstrated a smaller contribution of eddy diffusion if compared to FP counterparts. Another important point is that in chiral chromatography an additional source of band broadening must be considered, that is adsorption-desorption kinetics. This term is usually neglected in achiral RP separations (except in the case of large proteins) since kinetics is very fast. Oppositely, it can be one of the main factors contributing to poor kinetic performance in chiral chromatography [20,21]. In particular, the authors suggests the importance of a deeper investigation of the impact of bonding density of chiral selector, base silica characteristics, surface heterogeneity, etc. on the adsorption-desorption kinetics [4,22,23].

However, development in column technology, on the one hand, and instrumental design, on the other, have permitted to decrease analysis time in chiral chromatography from tenths of minute to less than one second (ultrafast enantioseparations). The key point has been to pack columns of very short length (5–20 mm long) with these new generation CSPs (be either SPPs or sub-2 μm FPPs) and operate them at the highest flow rate achievable by state-of-the-art equipments.

The scope is this review is to give an overview of the evolution of chiral chromatography towards ultrafast enantioseparations of chiral bioactive molecules in the last three years. Not only the recent achievements in the field of LC will be reviewed but also those obtained in supercritical fluid chromatography (SFC), where new generation CSPs have been more recently introduced. Particular attention will be also focused on instrumental modifications required on chromatographic equipments in order to fully exploit the intrinsic potential of new generation chiral columns.

## 2. Theory Background

Separation efficiency is conventionally evaluated in terms of number of theoretical plates, *N*, or plate height, *H*:

(1) $$H=\frac{L}{N}$$

being *L* the length of the column. The smaller *H* (higher *N*), the higher the efficiency.

In order to compare the efficiency of columns packed with different particle geometries, instead of *H*, its adimensional quantity, the reduced plate height, *h*, can be used. *h* is defined as follows:

(2) $$h=\frac{H}{d_{p}}$$

being $d_{p}$ the mean diameter of the particles forming the packed bed. Since inside particle there is absence of flow, the right velocity to refer to, when evaluating kinetic performance, is the interstitial velocity, $u_{e}$:

(3) $$u_{e}=\frac{F_{v}}{\pi r_{c}^{2}\epsilon_{e}}$$

where $F_{v}$ is the flow rate, $r_{c}$ the radius of the column and $\epsilon_{e}$ the interstial porosity (referred only to the portion of column where there is effectively flow). The adimensional quantity used to express mobile phase velocity is the reduced velocity, ν:

(4) $$\nu =\frac{u_{e}d_{p}}{D_{m}}$$

where $D_{m}$ is the molecular diffusion coefficient of the analyte in the bulk mobile phase. *h* and ν are correlated by means of the well known van Deemter equation:

(5) $$h=a\left(\nu \right)+\frac{b}{\nu }+c_{s}\nu +c_{ads}\nu +h_{extra}+h_{heat}$$

where a(ν) represents the contribution to band broadening of eddy dispersion, *b* is the longitudinal diffusion, $c_{s}$ the solid-liquid mass transfer resistance, $c_{ads}$ the adsorption-desorption kinetics, $h_{extra}$ extra-column band broadening and $h_{heat}$ an additional source of band spreading due to frictional heating effects between the eluent at high flow velocity and packed beds made of very fine particles (such as sub-2 μm FPPs).

One of the main approaches used to increase column efficiency is to reduce particle diameter since *H* is proportional to 2$d_{p}$. However, the main drawback of the use of such fine particles is that significant backpressure can be generated by the packed bed even at moderate flow rates, so that columns packed with these particles can be only used on Ultra-High Performance (or Pressure) Liquid Chromatography (UHPLC) instruments able to reach up to 1200–1500 bars. In 2006 SPPs have been introduced. The presence of the solid core reduces the volume accessible for analytes to diffuse inside the particle. As a consequence, it has been demonstrated that columns packed with sub-3 μm SPPs produce, at least in achiral RPLC, efficiencies comparable to those of sub-2 μm FPPs but at significantly lower backpressure [4,24,25,26,27]. However, mass transfer contributions in chiral chromatography have been barely investigated before, mainly due to the complexity of estimation of each band broadening contributions. Deeper studies are especially needed in order to quantify the effect of $c_{ads}$ on column efficiency [4].

## 3. Recent Achievements in Ultrafast, High-Throughput UHPLC Enantioseparations

In this section the most important results in terms of ultrafast, high-throughput enantioseparations obtained in UHPLC conditions will be discussed. For the sake of simplicity, applications have been divided according to the type of particle used.

### 3.1. CSPs Based on FPPs

As mentioned before, the first example of new generation CSPs has been developed by Gasparrini and co-workers by bonding a Pirkle-type chiral selector to sub-2 μm FPPs [8]. The choice of this class of chiral selector to start the transition from enantioselective HPLC to UHPLC finds its rationale on the ease of functionalization of even small particles (absence of particle aggregation and clogging during their synthesis) and on the supposed fast mass transfer kinetics properties of CSPs based on this class of selectors [11,28].

The same group has obtained the enantioseparation of *trans*-stilbene oxide enantiomers in 0.9 s on a 10 × 3.0 mm (L × I.D.) column packed with 1.8 μm FPPs functionalized with a Pirkle-type (more specifically, Whelk-O1) chiral selector. Flow rate was set at the maximum value deliverable by the equipment (8 mL/min) [6].

**Figure 1:**
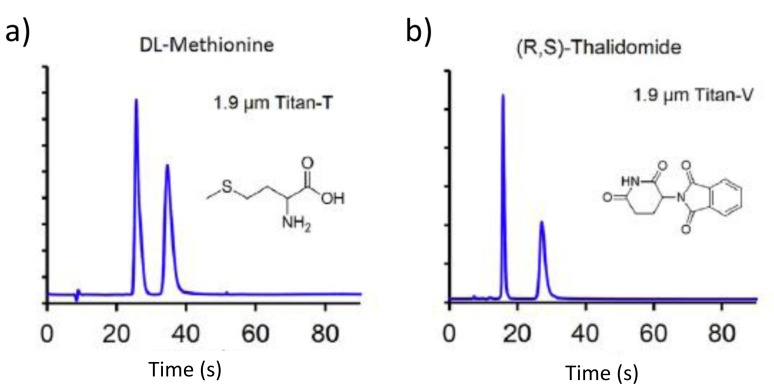
Examples of fast separations on 50 × 4.6 mm (L × I.D.) columns packed with nPSD 1.9 μm FPP CSP. (**a**) chiral selector: teicoplanin, compound: DL-methionine, mobile phase: water/methanol 30%:70% (*v*/*v*), $F_{v}$ = 2 mL/min; (**b**) chiral selector: vancomycin, compound: (R,S)-thalidomide, mobile phase: 100% methanol, $F_{v}$ = 3 mL/min. Modified with permission from reference [29].

Barhate et al. found efficiencies up to 210,000 N/m with columns packed with 1.9 μm Titan${}^{®}$ particles characterized by a narrow particle size distribution (nPSD), with a reative standard deviation smaller than 10%, functionalized with teicoplanin, teicoplanin aglycone and vancomycin. They were able to separate different classes of bioactive chiral compounds including aminoacids, β-blockers and pharmaceuticals with analysis time in some cases smaller than 40 s on 50 × 4.6 mm (L × I.D.) columns (see Figure 1) [29].

Ismail et al. recently employed a proprietary bonding protocol to obtain the zwitterionic version of teicoplanin and vancomycin CSPs bonded to 1.9 μm Titan${}^{®}$ FPPs [30]. Thanks to the zwitterionic character of these phases, chiral Active Pharmaceutical Ingredients (APIs) have been separated from inorganic anions, which are usually unretained or even excluded from traditional versions of macrocyclic glycopeptide CSPs, due to electrostatic repulsion. Moreover, the same authors obtained the separation of Haloxyfop enantiomers in 4 s ($F_{v}$ = 8 mL/min) on a 20 × 4.6 mm (L × I.D.) column packed with the 1.9 μm FPPs zwitterionic teicoplanin CSP [5]. The introduction of these particles has rekindled the debate about the possible correlation between column efficiency and PSD. Until 1970’s, common belief was that, unless PSD is larger than 40%, its influence on column performance is negligible [31,32,33,34]. More recently, some authors found that PSD has an effect on column efficiency as long as its RSD ranges from 5% to 20% [35,36]. As a marginal remark, excellent kinetic performance ($h_{min}$ = 1.7 corresponding to an efficiency of 300,000 N/m) has been also observed for the separation of achiral compounds on columns packed with C${}_{18}$ Titan${}^{®}$ particles [6,7,37,38].

The growing interest in high-throughput analysis has led to the development of solutions able to overcome the instrumental limitation imposed by autosamplers, whose typical injection cycle time is 20 s or even more. One of the most used approaches is MISER (Multiple Injections in a Single Experimental Run), whcih allows not only to reduce analysis times but also to produce a repetitive chromatogram that facilitates the interpretation of results when large number of samples must be analyzed. A 5 × 4.6 mm (L × I.D.) column packed with 1.9 μm nPSD particles functionalized with vancomycin chiral selector has been used for the MISER analysis of enantiopurity of the drug thalidomide with injection cycle time of only 10.5 s. This approach can be used for the analysis of an entire microwell plate in very short times [39].

Columns packed with sub-2 μm chiral FPPs have been demonstrated to be suitable for the use as second dimension for 2D achiral × chiral separations. Due to the slowness of elution, the employment of chiral columns for this type of analysis has always been very limited in the past. On the other hand, Barhate et al. excellently employed a 50 × 4.6 mm (L × I.D.) column packed with 1.9 μm teicoplanin FPPs for the baseline resolution of two isomers coeluting in the first achiral dimension [40].

Remarkably, some authors have also obtained very fast separations with FPPs with dimensions higher than sub-2 μm. Khundadze et al. were able to separate chiral sulfoxide derivatives in less than 3 s ($F_{v}$ = 4.5 mL/min) on a 10 × 2.0 mm packed with 3 μm phenylhexyl FPPs to which a cellulose derivative was bonded. In this specific case, temperature was increased up to 70 ${}^{\circ }$C in order to improve mass transfer and decrease retention times of the second eluted enantiomer [41].

A 5 μm FPP cellulose-based CSP was packed into the first example of microfluidic pressure-driven separation device that allowed the separation of different racemic mixtures, including also pharmaceuticals such as fluvastatin and rimadyl extract, under different chromatographic conditions. Very short separation times down to 5 s were achieved with reduced plate of about 2.2 on a 12 mm long channel [42].

### 3.2. CSPs Based on SPPs

The most comprehensive works aimed at evaluating the potential of sub-3 μm SPPs towards ultrafast, high-throughput enantioseparations are those published by Armstrong’s group. In a first report of 2015, they functionalized 2.7 μm SPPs with different chiral selectors (including teicoplanin, cyclofructans and cyclodextrins). These CSPs were then packed into columns of 50 or 100 mm length and operated under different chromatographic modes (i.e., RP, HILIC and polar organic mode to name but a few) to separate 60 pairs of enantiomers. Separations in the range of 4–40 s have been obtained [2]. Moreover, the same CSPs have been used to separate fluorinated APIs from their desfluoro analogs with analysis times smaller than 55 s in both reversed phase and polar organic mode [15].

Fast separations (under 1.5 s) of chiral sulfoxides have been obtained by Chankvetadze’s group by using a 20 × 2.0 mm (L × I.D.) column packed with 2.6 μm SPPs functionalized with a cellulose derivative [41].

Analysis times have been recently further decreased under the second time scale and examples of ultrafast enantioseparations using sub-3 μm SPPs have been reported by different authors. *Trans*-stilbene oxide enantiomers have been separated in 0.82 s ($F_{v}$ = 8 mL/min) on a 10 × 3.0 mm (L × I.D.) column packed with Whelk-O1 2.6 μm SPPs by our research group [4]. Sub-second separations ($F_{v}$ = 7.5 mL/min) of lorazepam and oxazepam enantiomers have been obtained by Armstrong and coworkers on a 10 × 3.0 mm (L × I.D.) column packed with 2.7 μm SPPs [43]. The same research group reported also on the enantioseparation of different biologically active compounds on 5 mm columns packed with 2.7 μm SPPs CSPs. Analysis times as low as 0.66 s have been obtained with a flow rate of 5 mL/min [17].

Columns packed with 2.7 μm SPPs functionalized with teicoplanin have been demonstrated to be suitable for the enantiopurity analysis of the entire verubecestat route [18]. Verubecestat is used in the treatment of Alzheimer’s disease. The analytical technical package of validated methods currently in use to evaluate the enantiomeric excess during its production requires two separation modes (both LC and SFC), five different chiral columns and four different mobile phases. Barhate et al. developed an alternative method in which analysis are performed only in RPLC mode. The enantiopurity of all intermediates was assessed by using a 2.7 μm teicoplanin SPPs CSP while another macrocyclic glycopeptide column made on the same particle format was found to be the most suitable for the analysis of the final API. This new approach, requiring only two chiral columns and mobile phases on a conventional HPLC system, simplifies the transfer to manufacturing facilities, significantly reducing waste of time.

A 100 × 4.6 mm (L × I.D.) column packed with a novel CSP based on 2.7 μm SPPs functionalized with hydroxypropyl β-cyclodextrins was used to separate five β-blockers (carvedilol, alprenolol, acebutolol, nadolol and atenolol) in less than 1 min ($F_{v}$ = 1 mL/min) under HILIC conditions [44].

**Figure 2:**
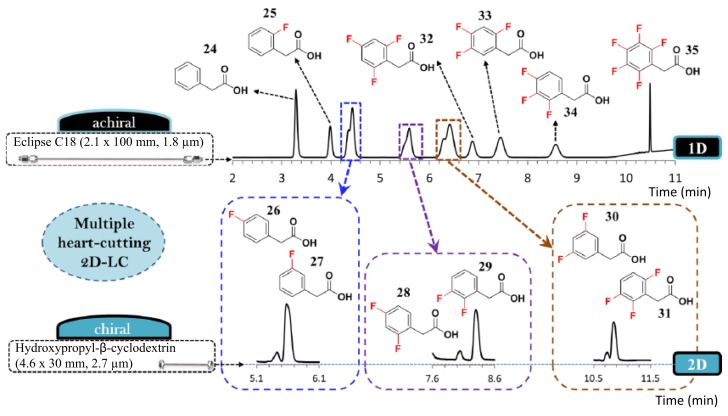
Multiple heart-cutting 2D-LC method for separation of a complex mixture of fluorophenylacetic acid isomers. Flow rates employed were $F_{v}$ = 0.5 mL/min for the first achiral dimension and 1 mL/min for the second chiral one. Modified with permission from ref. [40].

The same CSP packed into a 30 × 4.6 mm (L × I.D.) column was used for achiral × chiral 2D separations [40]. The use of a fast (separation times in the range of 26–52 s) and efficient chiral column as second dimension allowed to perform multiple heart-cutting analysis of fluorophenylacetic acid isomers (see Figure 2). This technique is different from the common comprehensive 2D-LC, where the entire first dimension is injected into the second one. In multiple heart-cutting mode only few selected portions of the first dimension chromatogram are cut and analysed in the second dimension. Each cut is stored into a loop system while waiting for the second dimension to be available for the next injection. This approach has been also employed by Lämmerhofer’s group in ref. [45] with an ion exchange chiral column made on 2.7 μm SPPs as second dimension to analyze 25 amino acids (20 of which were proteinogenic) in a single run with 130 min total run time.

The same group recently developed a chiral × chiral 2D-LC method based on two columns packed with 2.7 μm SPPs functionalized with the same chiral selector but with different configuration (quinine and quinidine carbamates) [46]. By means of this approach, highly ordered chromatograms can be obtained that can be used to get important information about the stereochemistry of peptides or unknown complex samples (such as nonribosomal peptides and therapeutic proteins) during pharmaceutical development or impurity analysis.

Thanks to the improvements in particle manufacturing and technology, the reduction of particle size to favour higher efficiencies and faster separations has been sought also with SPPs. Very high efficiencies can be achievable with packed beds made of SPPs with diameter smaller than 2.6–2.7 μm at the cost of increased backpressure. In literature there are only few reports on the use of small SPPs in chiral chromatography, however it is expected that the number of papers will increase in the next future. Ismail et al. reported on the use of 2.0 μm SPPs functionalized with teicoplanin and operated in HILIC mode [5]. They were able to obtain the enantioseparation of haloxyfop enantiomers in 3.4 s ($F_{v}$ = 8 mL/min) with very high efficiencies ($h_{min}$ ≃ 1.7) on a 20 × 4.6 mm (L × I.D.) column packed with the novel teicoplanin CSP. Patel et al. performed ultrafast enantioseparations of different compounds on a 10 × 3.0 mm (L × I.D.) column packed with 1.9 μm SPPs functionalized with quinine. The simultaneous separation of DNB-phenylglycine and 2-phenylpropionic acid enantiomers has been obtained in less than 1 s ($F_{v}$ = 7.85 mL/min) [43]. Even smaller SPPs (1.5 μm diameter) functionalized with teicoplanin have been used by Min et al. for the enantioseparation of different pairs of amino acids achieving high resolution and selectivity [47]. For instance, norvaline enantiomers were separated with a resolution factor of 4 and a selectivity higher than 9.

### 3.3. Instrumental Limitations

When working with ultrafast, high efficiency enantioseparations, important disadvantages could come from instrumental limitations. The extra-column variance of the equipment must be reduced by using short and narrow tubings, low-volume detector cells (2–3 μL) and ultra-low dispersion injectors. Another important aspect to be considered is the speed of autosamplers. 15–20 s injection cycle times can represent an obstacles for high-troughput separations [3]. Due to increasing number of applications in which separation times are in the order of few seconds, new, faster autosampler need to be designed.

Also sampling frequency of diode array detectors is another parameter that needs to be optimized. If it is not appropriate, many signals might be lost. According to a recent study published by Wahab et al. [17], the maximum sampling frequency achievable on current instrumentations is high enough for commercially available high efficiency columns. However, due to the continuous improvements in design of new materials for ultrafast chromatography, it is predictable that higher sampling rates might be shortly required [48].

## 4. Advances in Ultrafast, High-Throughput SFC Enantioseparations

SFC has been largely used in the past for the purification of chiral compounds, due to its green character, versatility and ease of removal of carbon dioxide (CO${}_{2}$) from collected fractions. However, less than ten years ago, thanks to the introduction of latest generation SFC equipments and the improvement in particle technology, the attention of scientists has been moved towards the use of SFC for analytical purposes. In particular, thanks to the unique properties of supercritical fluid CO${}_{2}$, SFC allows to run faster separations than in LC without remarkable loss of efficiency and with reduced backpressure. For these reasons, SFC seems to be a promising technique in the field of high speed enantioseparations [49].

**Figure 3:**
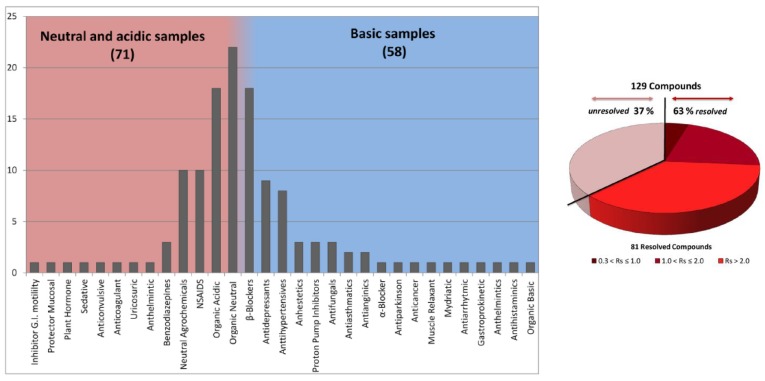
Classification of the different molecules analyzed during fast gradient screening (left); pie chart reporting numerical proportion of resolved enantiomers with different resolution values ($R_{s}$ = 2(t${}_{R,2}$ − t${}_{R,1}$)/(w${}_{0.5,2}$ + w${}_{0.5,1}$), being $t_{R}$ retention time and w${}_{0.5}$ peak width at half height. Modified with permission from ref. [51].

Different examples of sub-minute separations of different chiral pharmaceuticals (including chlortalidone and 5-methyl-5-phenylhydantoin) have been performed by Barhate et al. by using 50 × 4.6 mm (L × I.D.) columns packed with nPSD 1.9 μm FPPs functionalized with teicoplanin and teicoplanin aglycone [29]. Also some of the authors of this work reported on remarkable results in terms of fast separations in SFC by using both teicoplanin 1.9 μm Titan${}^{®}$ FPPs and Whelk-O1 1.8 μm FPPs packed into 20 and 50 × 4.6 (L × I.D.) columns, respectively [50,51]. The latter column has also been excellently used for the screening of a large library of compounds (including antidepressant, β-blockers and antibiotics, see Figure 3) under fast gradient elution conditions (total analysis time 9 min, including column re-equilibration) with a score of 63% positively resolved couples of enantiomers [51].

Even faster separations have been obtained by Armstrong’s group by running separations of different amino acids at a flow rate of 20 mL/min on a 30 × 4.6 mm (L × I.D.) column packed with 2.7 μm SPPs. Elution times in the range of 6–8 s have been observed [52].

### Instrumental Limitations

The achievement of ultrafast, high efficiency enantioseparations in SFC is currently limited by the excessively large extra-column volume of state-of-the-art SFC equipments. Technological advancements in SFC instrumentation have been much slower with respect to what happened in LC.

**Figure 4:**
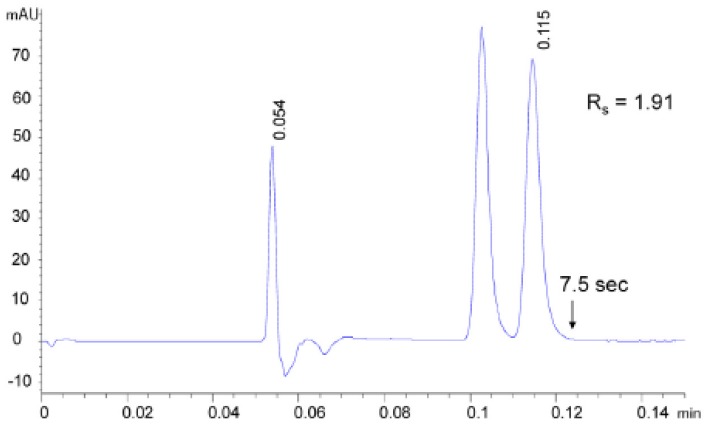
Fast SFC separation of *trans*-stilbene oxide enantiomers on a 50 × 3.0 mm (L × I.D.) column packed with 1.6 μm FPPs functionalized with amylose, $F_{v}$ = 4.65 mL/min, mobile phase: CO${}_{2}$/methanol 70%:30% (*v*/*v*), 40 ${}^{\circ }$C. Reproduced with permission from ref. [54].

Different authors have demonstrated that tremendous improvements in column efficiency could be achieved by properly optimizing the plumbing of commercially available SFC equipments. Berger firstly went through this issue by replacing tubings (170 μm ID) and flow cell (13 μL internal volume) with 120 μm I.D. tubings of shortest possible length and a 2 μL internal volume cell. With this setup, the extra-column variance was reduced up to 6–9 μL${}^{2}$ and fast enantioseparations (up to less than 8 s, see Figure 4) of different pharmaceuticals have been obtained with 50 mm long columns packed with both Whelk-O1 1.8 μm FPPs and amylose-based 1.6 μm FPPs, with efficiencies as high as 280,000 N/m [53,54].

More recently, Ismail et al. performed a series of technical adjustments on a commercial SFC instrument (of a different brand from that used by Berger) by replacing (i) tubings with narrower capillaries; (ii) the 8 μL flow cell with a 3 μL one; (iii) the injector with a 200 nL fixed-loop external one and (iv) the column oven with a in-house designed one. The latter adjustment, in particular, was crucial for the reduction of the extra-column variance by more than 97% (from 85 to slightly more than 2 μL${}^{2}$, measured at 2 mL/min) from the as-shipped configuration to the optimized one [55]. This has been possible thanks to the presence at even low flow rates (1.5–4 mL/min) of fully-developed turbulent flow regime inside capillaries of proper length and I.D. Unmatched kinetic performance of roughly 300,000 N/m have been obtained on a 100 × 3.0 mm (L × I.D.) column packed with Whelk-O1 1.8 μm FPPs.

## 5. Final Remarks

The introduction of very efficient particle formats, such as sub-2 μm FPPs and sub-3 μm SPPs, functionalized with chiral selectors has represented the most important innovation in the field of chiral chromatography towards ultrafast, high-throughput enantioseparations. However, the continuous evolution in particle design and material technology will lead lo the development of smaller and smaller particles which will require the availability of equipments able to tolerate very high pressures and with minimal extra-column volumes. Technical improvements are especially required on SFC instruments, since extra-column contribution of state-of-the-art equipment is too large with respect to variance of a very narrow peak eluted from high efficiency columns.

New generation CSPs are expected to be increasingly applied as chromatographic supports for miniaturized platforms and microchips and multidimensional applications. In parallel, faster autosamplers will be needed in order to perform very fast screenings.

From a more fundamental point of view, deeper studies of mass transfer phenomena in chiral chromatography are necessary in order to understand the impact of adsorption-desorption kinetics on column efficiency, its possible correlation with bonding denisty and if this term could respresent a kinetic limit to ultrafast enantioseparations.
